# Advanced Structural and Technological Method of Reducing Distortion in Thin-Walled Welded Structures

**DOI:** 10.3390/ma14030504

**Published:** 2021-01-21

**Authors:** Piotr Horajski, Lukasz Bohdal, Leon Kukielka, Radoslaw Patyk, Pawel Kaldunski, Stanislaw Legutko

**Affiliations:** 1 Faculty of Mechanical Engineering, Koszalin University of Technology, 75-620 Koszalin, Poland; piotr.horajski@gmail.com (P.H.); leon.kukielka@tu.koszalin.pl (L.K.); radoslaw.patyk@tu.koszalin.pl (R.P.); pawel.kaldunski@tu.koszalin.pl (P.K.); 2 Faculty of Mechanical Engineering, Poznan University of Technology, 60-965 Poznan, Poland; stanislaw.legutko@put.poznan.pl

**Keywords:** welding, distortion, thin-walled structures, structural and technological changes, advanced method, FEM, numerical calculation, heat transfer, welding stress

## Abstract

The article presents an innovative method of reducing welding distortions of thin-walled structures by introducing structural and technological changes. The accuracy of the method was demonstrated on the example of welding the stub pipes to the outer surface of a thin-walled tank with large dimensions, made of steel 1.4301 with a wall thickness of 1.5 × 10^−3^ (m). During traditional Gas Tungsten Arc Welding (GTAW), distortions of the base are formed, the flatness deviation of which was 11.9 × 10^−3^ (m) and exceeded the permissible standards. As a result of structural and technological changes, not only does the joint stiffness increase, but also a favorable stress state is introduced in the flange, which reduces the local welding stresses. Numerical models were developed using the finite element method (FEM), which were used to analyze the residual stresses and strains pre-welding, in extruded flanges, in transient, and post-welding. The results of the calculations for various flanges heights show that there is a limit height h = 9.2 × 10^−3^ (m), above which flange cracks during extrusion. A function for calculating the flange height was developed due to the required stress state. The results of numerical calculations were verified experimentally on a designed and built test stand for extrusion the flange. The results of experimental research confirmed the results of numerical simulations. For further tests, bases with a flange h = 6 × 10^−3^ (m) were used, to which a stub pipe was welded using the GTAW method. After the welding process, the distortion of the base was measured with the ATOS III scanner (GOM a Zeiss company, Oberkochen, Germany). It has been shown that the developed methodology is correct, and the introduced structural and technological changes result in a favorable reduction of welding stresses and a reduction in the flatness deviation of the base in the welded joint to 0.39 × 10^−3^ (m), which meets the requirements of the standards.

## 1. Introduction

In mechanical engineering, welding is most often used as inseparable connections of components. In the case of large-size welded steel structures, mainly of low stiffness, the basic problem is their deformation in the form of permanent displacements and deformations. The cause of deformation is the local welding stresses arising as a result of the impact of thermal phenomena occurring during welding. For each responsible engineering design, a Welding Procedure Qualification Record (WPQR) is developed along with an approved Welding Procedure Specification (WPS). Carrying out instructions requires a lot of knowledge and professional experience. When designing and producing large-size structures, prototypes are usually used. In such cases, it is not possible to perform tests or introduce changes to production. Therefore, design and technological errors cannot be allowed. In the case of designing structures, tools supporting the engineer’s work are commonly used in the form of Computer Aided Design/Computer Aided Engineering (CAD/CAE) tools (preparation of documentation and engineering calculations). However, CAE methods are rarely used to design technologies, especially those with a complex course of physical phenomena. The reason is that the construction of numerical models of technological processes is complicated and goes beyond the scope of typical engineering knowledge. Welding of elements is a particularly difficult process to design, because the connected parts are subjected to complex thermal phenomena and structural changes. The heat source, which may be a gas flame, an electric arc, a plasma beam, an electron beam, or a laser beam, moves along the welding line, intensely heating the weld area [1,2]. The movement of the heat source also causes uneven heating of the joined elements. The areas surrounding the joint are subjected to various heating up to the melting point of the material and then cooled down to the ambient temperature. Such large changes in temperature are accompanied by the phenomenon of expansion and contraction of the material. Temperature expansion of bodies causes stresses, displacements and welding deformations. Uneven temperature distribution and unsteady heat flow are the cause of different thermal and mechanical reaction of connected elements. Thermo-mechanical properties of metals influence the kinetics of stresses and thermal deformations, and consequently deformations of the structure [3,4]. Depending on the design solution and the developed technology of production of welded parts, both deformations and stresses will appear in different ways [5,6]. In the case of massive (rigid) structures made of thick elements, the welding deformations are small, while the stresses in the areas of welded joints can reach high values. On the other hand, low rigidity (flaccid and thin-walled) structures tend to distort after welding. In such cases, the forces resulting from thermo-mechanical phenomena are so great that they are able to overcome the stiffness of the elements and cause their deformation. However, the stresses in such structures are lower. Various constructional and technological methods can be used to reduce the adverse impact of welding stresses and deformations on the structure being produced. Currently, the most important methods of reducing welding distortion are [7,8,9,10,11,12,13,14,15,16,17,18]: pre-welding, in transient welding distortion, and post-welding distortion prevention method. As a rule, the pre-welding distortion prevention method uses properly designed welding guns. The methods of in transient welding distortion prevention method include preheating of the welding zone and adequate cooling after welding [3]. However, in Reference [19], the method of rolling welds (post-welding) is presented, which introduces favorable compressive stresses reducing welding stresses. This method can be used for rigid objects, but it is not suitable for slender objects, such as thin-walled large-size tanks with connectors. Reference [20] analyzes the influence of the rolling process of welded sheets that are subjected to the deep drawing process. The beneficial effect of introducing compressive stresses during the rolling mill after welding was demonstrated. The changes introduced in the surface layer of the welded area as a result of rolling allowed a significant reduction of deformation after extrusion. Gunnert [21] focuses on a specific residual stress measurement method and the releasing of residual stresses by drilling a ring groove around the measuring marks arranged in a star pattern. Malisius [22] analyzes the influence of residual stresses in terms of the risk of crack initiation.

Reference [23] presents the effect of post-weld heat treatment on the residual stress and deformation of the structure made of 20/0Cr18Ni9 steel. The method gives good results; however, it is difficult to apply in the analyzed case of a thin-walled tank structure, as it may lead to additional uncontrolled deformations. The possibilities reduction of welding residual stresses and deformations were analyzed also in the works [24,25]. Reference [26] analyzes the deformations formed after laser welding. The results of the effect of normalizing treatment on the deformations and microstructure formed have been presented. It has been shown that the use of heat transfer as a post-welding distortion prevention method gives good results and enables the reduction of welding stresses. Reference [27] presents the results of numerical analyzes validated experimentally during the welding process. It has been shown that the application of the finite element method (FEM) model enables effective design of welding technology with the use of thermal analyzes. Reference [28] presents an analysis of the influence of the welding process of steel sheets on the occurring thermal deformations. The analyzes were performed using FEM, which was verified experimentally. FEM analyzes showed high compliance with the real object, confirming that FEM is an effective method of modeling thermal phenomena arising during the welding process. Reference [29] presents a numerical method of predicting deformations in welded joints of thin sheets. High compliance of the numerical models with the experimental ones has been demonstrated, which allows us to conclude that thermal analyzes using FEM are a good tool for estimating the distortion of thin-walled structures after welding.

**Figure 1:**
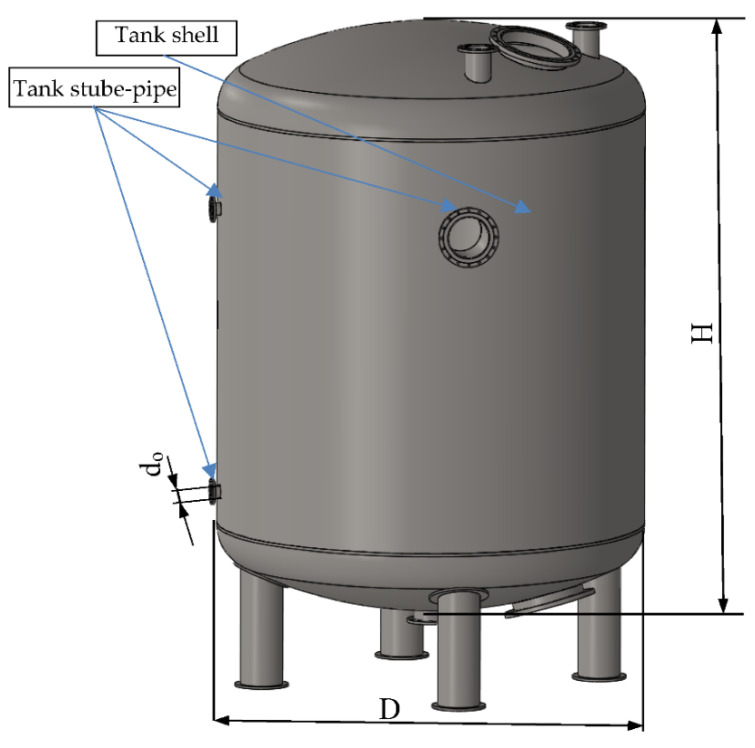
An example of a thin-walled tank for a liquid with stub-pipes.

Based on the literature analysis, it was found that there is a need for effective, cheap and simple methods of reducing deformations resulting from welding [30,31,32,33,34]. The described methods of reducing welding stresses are suitable for rigid structures, but are not suitable for reducing stresses in low-rigid structures [35,36]. The problem is particularly significant in the context of the production of large-size thin-walled welded structures with low stiffness. Therefore, the presented article concerns an innovative method of reducing welding deformation. It belongs to the pre-welding distortion prevention method and applies mainly to thin-walled and low-rigid structures. The essence of the proposed method is the introduction of structural and technological changes. First, favorable design changes are introduced that increase the stiffness of the structure. Then, technological changes are introduced, which at the same time additionally increase the rigidity of the structure and introduce a favorable stress state that reduces the stresses arising in the welding process. As a result, the deformation of welded structures, which reach the permissible level, is reduced. Both in the first and the second stage, computer-aided design using FEM and numerical methods is used. The method is presented on the example of a thin-walled tank to which connection stubs are welded (Figure 1). The analyzed tank is a flaccid shell structure, not resistant to point loads. However, it exhibits high stiffness and strength during even loading with internal pressure.

In the paper, an algorithm for reducing the deformation of the coating in the welding area was developed, which will be described on the example of a welded joint, which consists of a stub pipe welded, by means of a one-sided fillet weld, to the outer surface of a cylindrical tank with an outer diameter of D = 3.6 (m), height H = 4.7 (m), and a capacity of V = 50 (m^3^), made of austenitic steel 1.4301, with a wall thickness of t = 1.5 × 10^−3^ (m) and an opening with a diameter of d_0_ (Figure 1). During welding, the welding stresses generated cause an unacceptable deformation of the tank shell.

**Figure 2:**
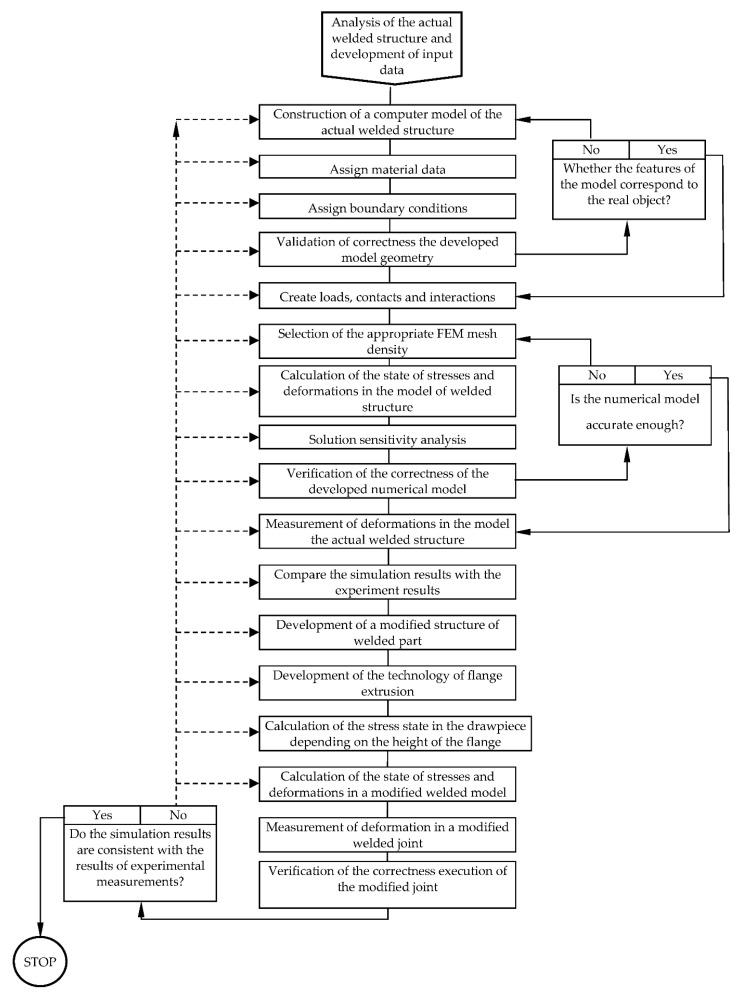
Flow chart of finite element analysis of coupled thermal and mechanical effects during welding of thin-walled structures.

A block diagram of the advanced constructional and technological method of distortion reduction in welded structures, using FEM, is presented in Figure 2.

### Problem Definition

**Figure 3:**
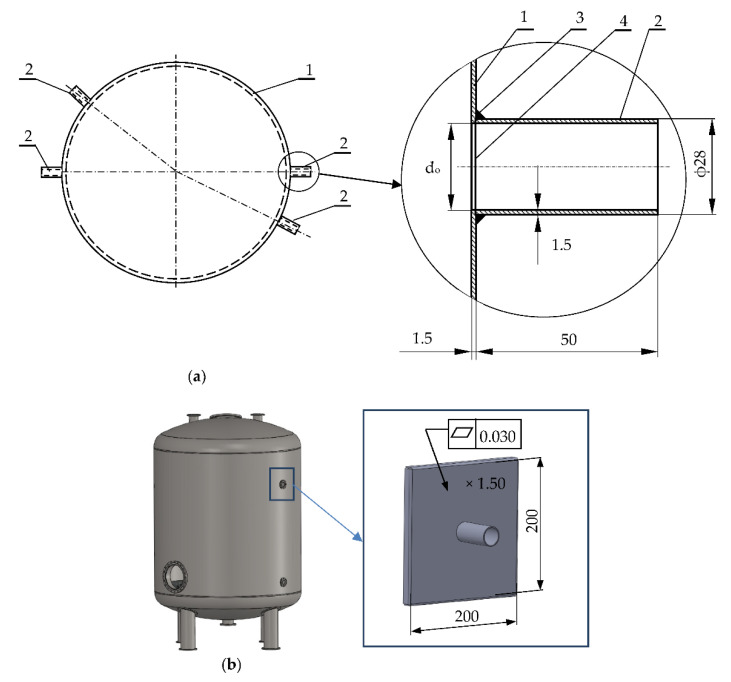
Welded tank structure: 1—cylindrical tank, 2—stub pipe, 3—one-sided fillet weld, 4—opening in the tank for the stub pipe (**a**), a test sample with modeled tank jacket area (**b**).

The problem to be solved in the presented article is the development of an innovative method of reducing welding deformations and checking it on the example of welding joints for large-size thin-walled tanks made of austenitic steel 1.4301. Currently, the Gas Tungsten Arc Welding (GTAW) welding of a stub-pipe to a quasi-flat shell surface with an opening without a flange causes its deformations leading to distortions within the heat-affected zone. The cause of the unfavorable phenomenon is the welding stresses occurring during welding, which cause unacceptable longitudinal, transverse, and thickness contractions. The proposed methodology is to prevent such unfavorable situations and reduce welding deformations to an acceptable level. The correctness of the developed calculation methodology was verified on the example of the designed welded joint. For this purpose, tests were carried out on appropriate samples. The surface of the cylindrical tank was replaced with its fragment in the form of a 0.2 × 0.2 (m) flat sample (Figure 3). The sheet plane was additionally strengthened by introducing bends at the edges. The sheet thickness for the analyzed case is t = 1.5 × 10^−3^ (m), while the dimensions of the stub pipe are ϕ 28 × 1.5 × 10^−3^ (m) and the height is h = 0.05 (m). Both elements are made of 1.4301 steel. The samples prepared in this way correctly model the stiffness of the tank shell structure. Before the actual samples were made, numerical analyzes and preliminary experimental tests were carried out in order to correctly develop adequate shapes and dimensions of samples for the actual tests. 

Austenitic steel 1.4301 containing in its chemical composition 17–19.5% chromium (Cr) and 8–10.5% nickel (Ni), in addition to corrosion resistance, shows high ductility, which makes it very susceptible to cold forming and welding. An important attribute is the ability to strengthen the material by means of properly developed cold working. Table 1 shows the physical and mechanical properties of EN 1.4301 steel at a room temperature of 293 °K.

**Figure 4:**
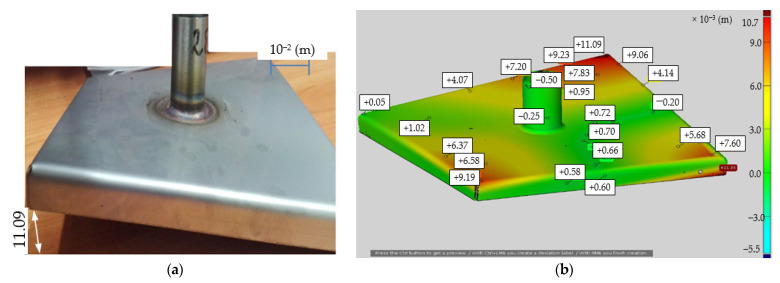
Test sample after TIG welding (method 141) (Gas Tungsten Arc Welding (GTAW)): photo of the real object (**a**), virtual model after 3D scanning with ATOS III scanner with measured deviation from the flatness values (**b**).

First, the stub pipe was welded to the base of the sample in the same conditions as when welding the stub pipe to the real tank. Welding was carried out using the TIG (Tungsten Inert Gas) method (method 141) (GTAW). The view of the sample after welding is shown in Figure 4a, while the virtual model obtained after 3D scanning with the ATOS III scanner (GOM a Zeiss company, Oberkochen, Germany) in Figure 4b. The presented figures show the maximum deviations from the flatness at the base corners of Δh ϵ (−0.7; 11.09) × 10^−3^ (m), which is unacceptable from the point of view of the tanks construction.

## 2. Numerical Solution of the Problem

The algorithm of proceeding according to the proposed innovative method of structural and technological reduction of welding distortions includes three main stages that must be performed in order to correctly solve the given problem:

(1) Development of a mathematical model of the welding process and the method of its solution. Then, a numerical calculation of welding displacements, strains, and stresses for traditional construction.
(2) Development of a model of an innovative structure increasing the rigidity of the system and development of a technology for manufacturing joint components. For this purpose, numerical models and methods of its solution should be developed, and then numerical calculation of stiffness, displacements, deformations, and residual stresses remaining in the structure after the technological process.
(3) Development a model of welding process of innovative structure with increased stiffness and a consciously shaped state of residual stresses. For this purpose, numerical models were developed to calculate the stress and deformation states in the welded joint, in which the proposed design and technological changes were applied. The correctness of the calculations were verified experimentally on special samples (Figure 3b).

In the next part, the individual stages of the procedure are presented, for example, by welding the socket to the base of the sample modeling the tank shell. The first stage of work was to develop an adequate numerical model of the welding process in order to determine deformations, strains, and stresses after the welding process. The second stage was the development of an adequate numerical model of the stiffening flange extrusion process, which, at the same time, introduces compressive stresses in the weld zone. The third stage was the development of an adequate numerical model of the welding process of a stub pipe with a stiffening flange. The obtained final result of the deformation state after the third stage makes it possible to predict the behavior of the real object.

### 2.1. Modeling of the Welding Process

The essence of modeling is to develop models of real systems, taking into account the required accuracy and acceptable modeling costs. Therefore, it is important to simplify the models as much as possible while obtaining correct simulation results. For modeling the welding process, due to the adopted aim (determination of the deformation state of the structure), only the analysis of the influence of thermal phenomena was undertaken. It is well known that when the temperature of the part increases, the value of plasticizing stress decreases, which, in extreme cases, can drastically reduce its stiffness. Therefore, the correct determination of the strength of a thermally loaded part requires knowledge, apart from the strength of materials, also of the thermodynamics of materials.

#### 2.1.1. Mathematical Model of Heat Movement

##### Incremental Differential Equation of Heat Transfer 

The analytical determination of the object’s temperature rise field during heating comes down to the solution of a nonlinear differential equation called the Fourier-Kirchhoff equation, for the general case, i.e., when there are moving heat sources and heat transfer occurs through conduction, convection, and radiation.

Nonlinearity of thermal phenomena requires the use of an incremental description. Therefore, an updated Lagrange description was adopted, which assumed knowledge of the temperature fields at the initial steps t_0_ and actual t, while a solution is found for the next step τ = t + Δt, where Δt is a very small increment of time. Then, the heat transfer incremental equation for a typical incremental step *t*→*τ*, in a fixed frame of reference {***z***}, for the case of mobile external heat sources, describes the transient temperature-rise fields in volumes of an object where heat is transferred by conduction. This equation has the form [37,38,39,40,41]:

(1) div{λtt(Ttt)⋅grad[ΔtτT(z,Δt)]}+Δtτq(⋅)=Ctt(Ttt)⋅ρtt(Ttt)⋅ΔtτT˙(z,Δt),

where:

(2) ΔtτT˙(z,Δt)=∂[ΔtτT(z,Δt)]∂t

is the rate of temperature rise, λtt(Ttt), Ctt(Ttt), ρtt(Ttt) are the temperature-dependent thermal conductivity, specific heat, and material density, respectively, at the beginning of the step, and Δtτq(⋅) is the increment in the efficiency of heat sources resulting from welding. 

An analytical solution of Equation (1), taking into account the initial condition and boundary conditions, consisting in determining the temperature-rise fields is not possible due to their entangled, complex form of the formulas for heat flux increases for a moving heat source and the dependence of thermophysical constants on temperature. However, an approximate numerical solution is possible. For this purpose, a variational formulation was used in which the developed functional is the total enthalpy of the system. Applying the stationarity condition of the functional, a variational equation of heat movement was obtained, which was discretized by the finite element method, resulting in a discrete equation of heat movement of the tested object.

##### Discrete Equation of Heat Motion

The object is divided by finite elements and the spatial discretization of the variational equation is performed. After the transformations, the discrete equation of heat motion is obtained in incremental form for a typical time step t→τ:

(3) [C t]{ΔtτΘ˙}+([K tk]+[K tc]+[K tr]+[K tIV]){ΔtτΘ}={ΔtτQ}+{ΔtτQI},

where [C t] is the global heat capacity matrix (at the beginning of the step), [K tk] is a global thermal conductivity matrix, [K tc] and [K tr] are global type III boundary condition matrices, convective (index c) and radiation (index r), [K tIV] is a global matrix of type IV boundary conditions, and {ΔtτQ} and {ΔtτQI} are global vectors on the step of heat load increment and type I boundary conditions, respectively.

##### Solving Discretized Equations of Heat Motion

The matrix Equation (3) is a system of N ordinary differential equations with constant (on an incremental step) coefficients. This equation is the basis of an approximate solution to the discussed problem. The solution is to calculate the temperature rise in the nodes of the object. Equation (3) contains twice as many unknowns as there are equations. In order to reduce the number of unknowns to the number of equations, the following approximation methods are used: Wilson, Newmark, Houbolt, or Newmark-Wilson [37,38,39,40,41]. These methods express the vector of temperature velocity increase in nodes {ΔtτΘ˙} by means of temperature rise in nodes ΔtτΘ. This transforms the system of differential equations into a system of algebraic equations. The use of these methods to approximate Equation (3) and the solution of the obtained algebraic equation system in order to calculate the temperature fields in the nodes of a discrete object are presented in the works [37,38,39,40,41]. In the exemplary approximation of the transient heat flow equation, the Houbolt method was used, in which the formula for the first derivative of temperature versus time is [42,43]:

(4) {Θ˙tτ}=a1{ΔtτΘ}−a2{Θtt}+a3{Θt−Δtt−Δt}−a4{Θt−2Δtt−2Δt},

where: a_1_ = 11/(6Δt), a_2_ = 7/(6Δt), a_3_ = 3/(2Δt), and a_4_ = 1/(3Δt) are integration constants.

By inserting expression (4) into the matrix Equation (3) and ordering it with respect to the variable ΔtτΘ the following equation is obtained:

(5) [K˜ t]{ΔtτΘ}={ΔtτQ˜}+{Q˜ t},

(6) where: [K˜ t]=a1[C t]+[K tk]+[K tc]+[K tr]+[K tIV],

(7) {Q˜ t}=[C t](a2{Θ t}−a3{Θ t−Δt}+a4{Θ t−2Δt}),

(8) {Δt τQ˜}={Δt τQ}+{Δt τQI}.

Effective matrix [K˜ t] in the discretized system of Equations (5) and an effective vector {Q˜ t} are known by design and assumed at the beginning of the incremental step. The component part of the vector {ΔtτΘ} is also known after taking into account the type I boundary conditions. And the vector {ΔtτQ˜} is unknown as it depends on the unknown temperature rise at the step considered. However, when using the iterative procedure, it is assumed that some of the components of the vector {ΔtτQ˜} is known from the previous iteration. Then, the nodes of the discrete model are divided into those in which: the heat load increase is given, and the temperature increase is unknown—*n* nodes, the temperature increase is given, and the heat load increase is unknown—*w* nodes.

Then, Equation (5) can be written in block form:

(9) [[K˜11nxn t][K˜12nxw t][K˜21wxn t][K˜22wxw t]]{{ΔtτΘ1nx1}{ΔtτΘ2wx1}}={{ΔtτQ˜1nx1}{ΔtτQ˜2wx1}}+{{Q˜1nx1 t}{Q˜2wx1 t}}.

Iterative solution of the system of equations in block I with respect to the unknown vector of temperature increase in nodes {ΔtτΘ1}**,** from the following system of equations obtained from (9):

(10) [K˜ t11]nxn[i−1]{ΔtτΘ1}nx1[i]={ΔtτQ˜1}nx1[i−1]+{Q˜ t1}nx1−[K˜ t12]nxw[i−1]{ΔtτΘ2}wx1.

Then, substituting the resulting vector {ΔtτΘ1} to the equation:

(11) {Δ tQ˜2}wx1[i]=[K˜ t21]wxn[i−1]{ΔtτΘ1}nx1[i]+[K˜ t22]wxw[i−1]{ΔtτΘ2}wx1−{Q˜ t2}wx1,

the unknown load increment vector in the *i*-iteration is obtained. After which, inserting {ΔtτΘ} to formula (4) the vector is obtained {ΔtτΘ˙}. A special startup procedure is used for the time *t* = *t_o_*.

##### Thermal Strength Calculations

**Figure 5:**
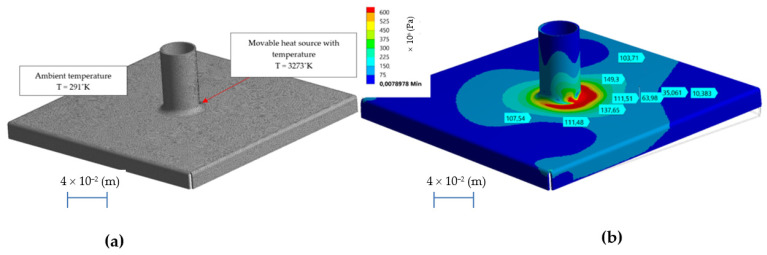
Discrete model of a test sample with added initial and boundary conditions (**a**), the state of effective stress after the welding process (**b**).

The methodology presented in Section 2.1.1 was used to calculate the temperature when welding the socket to the base of the sample, shown in Figure 3b. During the welding process, the material is locally heated up to the temperature of T = 3273 °K and quick cooled to ambient temperature T = 291 °K. Phase transformations and welding stresses cause deformation of the structure. The discrete model of the research object, containing NE = 154,802 finite elements of SOLID type, and NN = 291,954 nodes with added boundary and initial conditions are shown in Figure 5a, while the simulation results are shown in Figure 5b.

### 2.2. Introducing Design and Technological Changes

The developed numerical model makes it possible to calculate the stress state and deformation of the structure after welding. In the analyzed case, it was confirmed that the stresses caused by the welding process in the primary structure exceed the value of plasticizing stresses and lead to uncontrolled deformations of the structure (σ_Y_ < σ_R_). For the analyzed case, the maximum deviation from the flatness are Δh = −8.5 × 10^−3^ (m), which is a value similar to that observed in the experiment.

In order to increase the stiffness of the sample structure, a structural and technological change was introduced for the analyzed case, replacing the existing hole in the shell with a flange of an appropriate height. Such a solution will increase the stiffness perpendicular to the base surface. Plastic extrusion of the flange increases the stiffness and additionally introduces favorable compressive stresses caused by circumferential stretching of the hole. It is assumed that the stresses introduced in the flange will reduce the welding stresses and, consequently, lower the deformation of the welded joint to an acceptable value.

#### Modeling of the Construction Process Increasing the Rigidity of the System-Flange Extrusion

**Figure 6:**
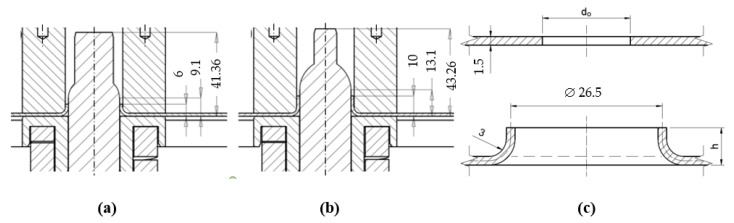
Cross-sections of the tools used for the circumferential stretching of the holes: a flange forming tool h_1_ = 6 × 10^−3^ (m) high (**a**), a flange forming tool h_2_ = 10^−2^ (m) high (**b**), and theoretical geometrical parameters of a shaped flange (**c**).

In order to determine the required height of an extruded flange to produce the desired stress state, the relationship of the stress state to the flange height was first determined. Then, as a result of solving the inverse problem, the required height of the flange was determined. A cross-section view of the designed exemplary flange extrusion tools with a height h_1_ = 6 × 10^−3^ (m) (named model h6) and height h_2_ = 10^−2^ (m) (named model h10), is shown in Figure 6.

Knowing the previously adopted assumptions, it is possible to calculate the approximate value of the initial diameter of the hole d_o_. For the theoretical flange height h_1_ = 6 × 10^−3^ (m), the hole diameter carries out d_0_=19.225 × 10^−3^ (m), and for the theoretical flange height h_1_ = 10^−2^ (m)-the value d_0_ = 11.225 × 10^−3^ (m). From the calculated value to on the basis of [44,45,46,47], the theoretical wall thickness t of the drawn flange was calculated. For h_1_ = 6 × 10^−3^ (m) and r = 1, the result was t = 1.278 × 10^−3^ (m), while for h_2_ = 10^−2^ (m) and r = 1, t = 0.976 × 10^−3^ (m). In order to assess the stress state in the product after the process of shaping the reinforcement flange and to assess the possibility of shaping the reinforcing flange, computer simulations of the process were performed. Numerical analyzes were performed using FEM. The drawing process was considered as a geometrically and physically nonlinear boundary-initial problem, in which there are nonlinear, moving and variable in time and space boundary conditions. The boundary conditions in the contact areas of the tool with the workpiece are unknown. The nonlinearities of the process and the high complexity of physical phenomena, with the lack of knowledge of the boundary conditions in the area of tool contact with the workpiece, require the use of modern modeling and analysis methods.

The mathematical description of nonlinear phenomena requires the use of other than in linear problems, the principles of formulating boundary-initial problems and more complex methods of solving them. In this paper, the updated Lagrange description was adopted for the description of the machining process at a typical incremental step. According to this description, at the current time t, the initial configuration of the body C 0 and the current configuration C t are known. This means that at these times and at all other times t<τ values of all functions occurring in the problem are known. Instead, the next equilibrium configuration C t+Δt, at the next time τ=t+Δt is found. By writing specific equations of motion for all finite elements separated from the tool and the workpiece, after their summation, the equation of motion of the technological process is obtained. The general equation of motion of a discrete object, in the updated Lagrange description, at a typical incremental step t→τ, has the form [37,38,39,40,41,46,47,48]:

(12) $$M\cdot \Delta \ddot{r}+C\cdot \Delta \dot{r}+\left(K+\Delta K\right)\cdot \Delta r=\Delta R+\Delta F+F+R,$$

where: M—instantaneous mass matrix of the system, C—instantaneous damping matrix, K and ΔK—instantaneous stiffness matrix and its increment, respectively, F, ΔF—instantaneous a of internal loads of nodes and its increment, R, ΔR—vector of surface loads and its increment, respectively, Δr—vector of node displacement increments, $\Delta \dot{r}$—node velocity increment vector, and $\Delta \ddot{r}$—node acceleration increment vector.

Matrix Equation (12) being a system of N second-order differential equations with constant (on an incremental step) coefficients with appropriate initial conditions $\left\{r\left(t=0\right)\right\}=\left\{r_{0}\right\},\;\left\{\dot{r}\left(t=0\right)\right\}=\left\{{\dot{r}}_{0}\right\}$ and boundary are the incremental formulation of the dynamic equilibrium of the deformed solids in contact, for the analyzed case. The system of Equation (12) contains N equations with N known elements of the vector of internal forces F and $3N^{2}$ elements of matrices M, C**,** and K, while 4N of unknowns, i.e., vector components: displacement increase Δr, node velocity increase $\Delta \dot{r}$, the acceleration increase of nodes $\Delta \ddot{r}$, and the increase in internal loads of the object ΔF and $N^{2}$ unknown elements of the matrix of the stiffness increase of the object ΔK. In this equation, also part of the components of the vector of the increase in external loads ΔR (in the contact areas) is unknown. In this paper, to solve the equation of motion, its partial linearization and explicit and implicit approximation methods were used. In the partial linearization procedure of the nonlinear incremental Equation (12), it is assumed that the time increment ∆t is very small. Then, it is possible to assume that the increase of the object stiffness matrix ΔK and the increase of the vector internal loads of the object ΔF are negligible. In this way, N + N^2^ unknowns are eliminated, and Equation (12) takes the form:

(13) $$M\cdot \Delta \ddot{r}+C\cdot \Delta \dot{r}+K\cdot \Delta r=F+\Delta R+R.$$

In the system of Equation (13) there are 3N unknowns, i.e., vector components: displacement increment of nodes Δr, node velocity increment $\Delta \dot{r}$, node acceleration increment $\Delta \ddot{r}$. In addition, some of the components of the vector of the increase in external loads ΔR (i.e., concerning the contact areas of bodies) is unknown. The obtained equation is further nonlinear with respect to the vector of the displacement increment of nodal points Δr and its time derivatives $\Delta \ddot{r},\;\Delta \dot{r}$. Next, the known methods of explicit and implicit approximation [42,43] were used, expressing the increases in the velocity of displacements and accelerations$\;\Delta \ddot{r},\;\Delta \dot{r}$ by means of the displacement increment Δr, obtaining the form of an equation from which the sought vector of increment in displacement of nodal points was determined.

The elastic/visco-plastic Cowper-Symonds model was used to model the yield stress σ_Y_ of the material in the process of shaping the flange during radial stretching of the hole. Huber-Mises-Hencky‘s yield criterion and the associated law of material flow were utilized in this model. A Cowper-Symonds model allows for linear-isotropic (β=1) or kinematic (β=0) plastic strain hardening and the effect of the intensity of plastic strain rate, in accordance with a following power relation:

(14) $$\sigma_{Y}={\left[1+\left({\dot{\varphi }}_{z}^{\left(p\right)}/C\right)\right]}^{m}\left(R_{e}+\beta \cdot E_{p}\cdot \varphi_{z}^{\left(p\right)}\right),$$

where:

β—is the parameter of plastic strain hardening,

R_e_—is the initial, static yield point (Pa),

${\dot{\varphi }}_{z}^{\left(p\right)}$—is the intensity of plastic strain rate $\left(s^{-1}\right)$,

C—is the material parameter defining the effect of the intensity of plastic strain rate $\left(s^{-1}\right)$,

m=1/P—is the material constant defining the material sensitivity to the plastic strain rate,

$\varphi_{z}^{\left(p\right)}\left(-\right)$—is the plastic strain intensity, and

$E_{p}=\frac{E_{T}\cdot E}{E-E_{T}}$—is the material parameter depending on the modulus of plastic strain hardening $E_{T}=\partial \sigma_{p}/\partial \varphi_{z}^{\left(p\right)}$ and Young’s modulus E. 

**Figure 7:**
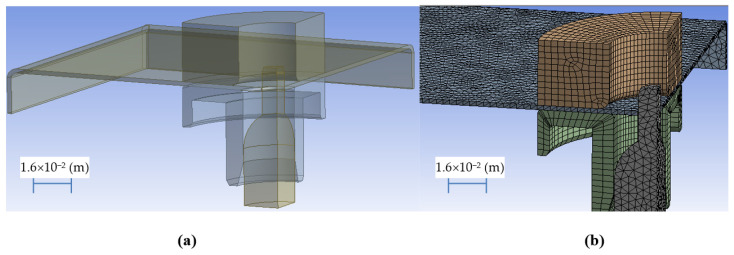
Geometric model (**a**) and discrete model (**b**) during the flange forming process-radial stretching of the hole.

Appropriate discretization is an important step in modeling the flange forming process during radial hole stretching for the three-dimensional stress state and the three-dimensional state of deformation. This is especially very important in the contact zones. Inadequate meshing causes the penetration of tool and sheet elements and insufficient representation of the material extrusion phenomenon. Too high density of the mesh causes a significant increase in computation time. Figure 7 presents a geometric model and an adequate discrete model (the effectiveness of the model was checked on the basis of a sensitivity analysis) of the process of shaping the flange during radial stretching of the hole.

**Figure 8:**
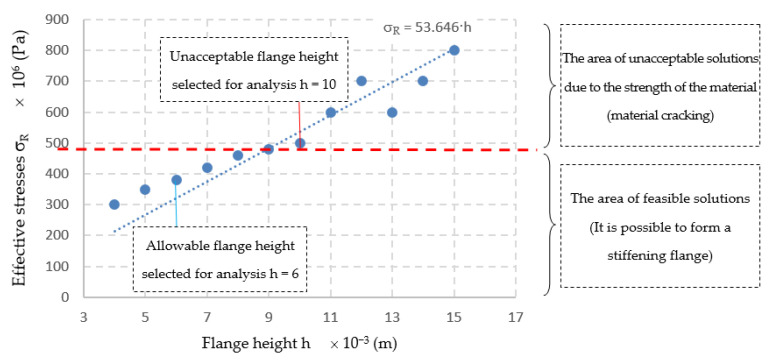
The relationship between effective stress and the flange height determined numerically.

After performing numerical calculations [46,47,48,49], the relationship was developed $\sigma_{R}=\;f\left(h\right)$ stresses from the flange height, shown in Figure 8, and the developed equation has the following form:

(15) $$\sigma_{R}=\;53.646\cdot h.$$

**Figure 9:**
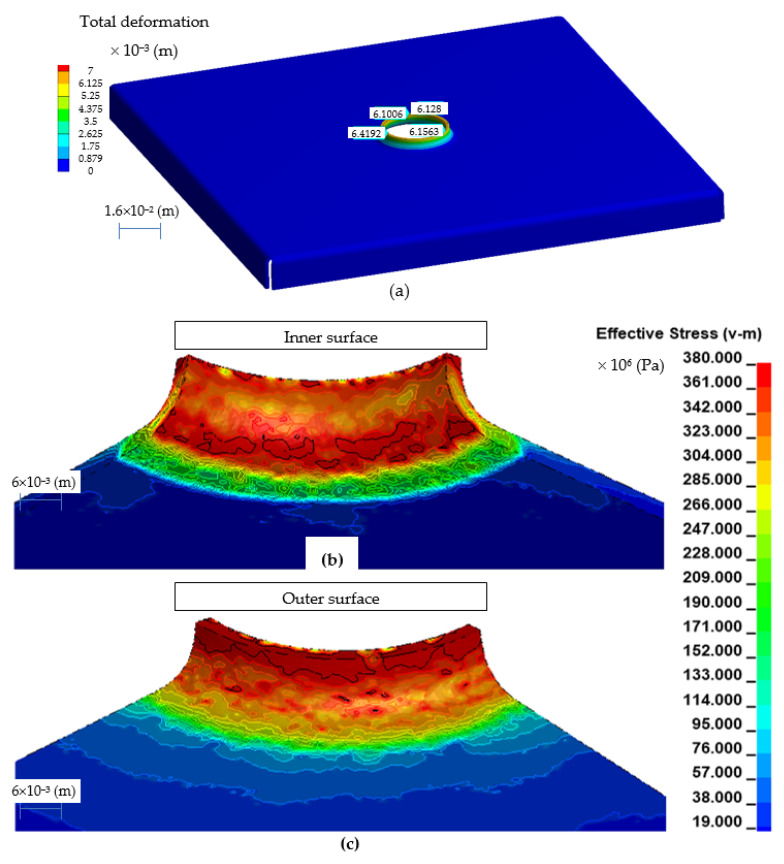
Effective stress in the embossed flange with a punch for the h6 model: view of the entire model (**a**), view of the inside side (**b**), and view of the outside side (**c**).

**Figure 10:**
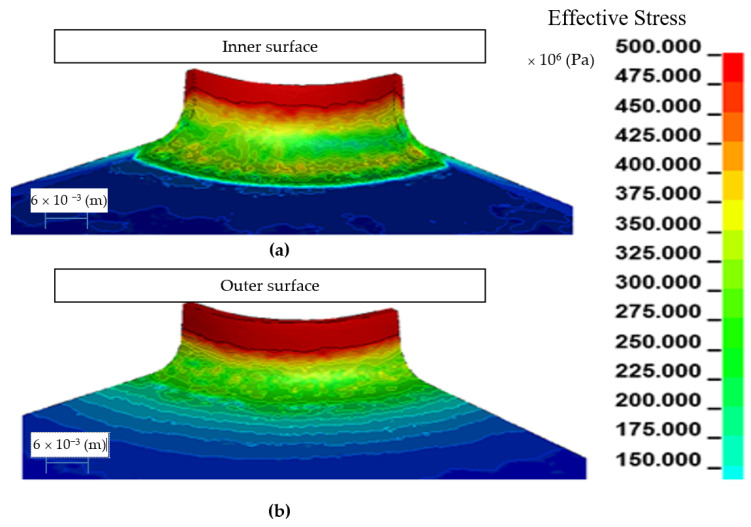
Effective stress in the embossed flange with a punch for the h10 model: view of the inside side (**a**), view of the outside side (**b**).

Example results of numerical calculations for the h6 model are shown in Figure 9, and for the h10 model in Figure 10.

The effective stress for the h6 model (Figure 9) reach the maximum value of 380 × 10^6^ (Pa) in the upper part of the flange, where the greatest change in the thickness of the sheet took place, to a value of about 1.24 × 10^−3^ (m). The effective stress for the h10 model (Figure 10) reach the maximum value of 556 × 10^6^ (Pa) in the upper part of the flange, where the greatest change in the thickness of the sheet took place, to a value of about 1.04 × 10^−3^ (m). On the basis of the conducted analyzes, it can be concluded that the h10 model obtains high values of effective stress and for the analyzed material-austenitic 1.4301 steel, there is a risk of tearing the shaped flange already during the manufacturing process.

The developed relationship of stresses on the height of the flange σ_R_ = f(h) (Figure 8) allows to determine the required height of the extruded flange, in which the state of stress corresponds to the welding stress. As a result of the superimposition of these stresses, the resultant stresses are significantly reduced to the required level (reverse task). For example, in the analyzed case, the height of the flange should be h = 6 × 10^−3^ (m), ensuring the correctness of the flange manufacturing process and ensuring reduction of deformation after welding.

### 2.3. Modeling the Welding Process of the Structure Increasing the Rigidity of the System

The next step, according to the algorithm of the structural and technological method of reducing deformation in welded structures, is modeling the welding process of the stub pipe to the flange, increasing the rigidity of the system. For further analysis, as a result of solving the inverse problem, the h6 model was selected because the h10 model showed too high initial stresses.

**Figure 11:**
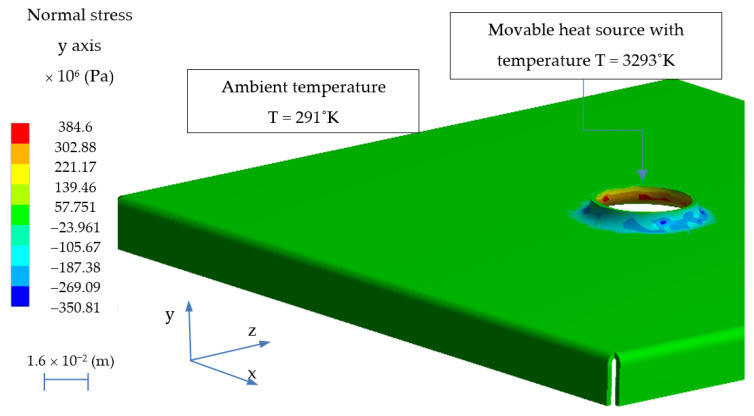
The state of normal stresses after the h6 flange extrusion process with thermal conditions originating from the welding process.

The modeling of the welding process of pre-stress structures consisted in the calculation of coupled fields. From the model of the flange extrusion process, the state of deformation, stress and strain was imported and after the extrusion process, and then it was heat loaded using a movable heat source at a temperature of T = 3273 °K (Figure 11).

**Figure 12:**
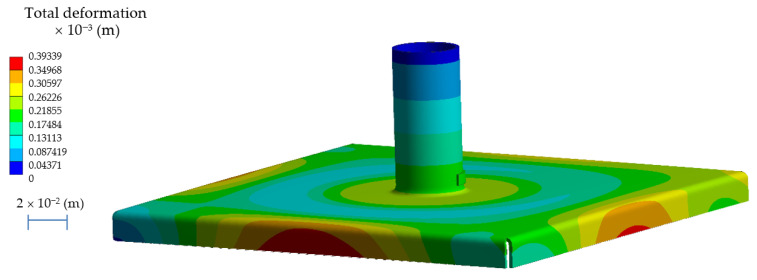
The state of deviation after the h6 flange extrusion process and stub welding.

After carrying out the calculations in accordance with the methodology presented in Section 2.1 and Section 2.2, the results of the state of displacements were obtained after the welding process of the new structure with a flange increasing the rigidity of the system. The calculated state of deviations is shown in Figure 12. The maximum deviations for such a model carry out Δh = 0.39 × 10^−3^ (m), which is a satisfactory value for industrial practice. The developed methodology of solving the given problem using numerical methods shows that it is possible to significantly improve the quality of the product. In order to verify the developed models and the methodology of the procedure, an experimental verification was carried out.

## 3. Experimental Studies of the Flange Drawing Process

**Figure 13:**
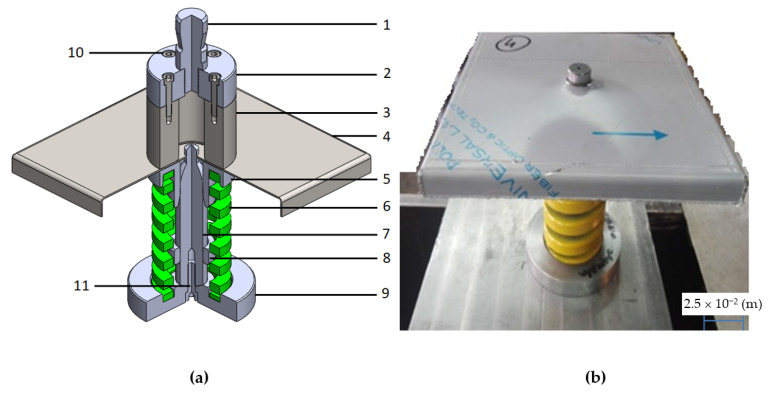
Geometric model of the tool for extrusion of the flange (**a**) and a photo of the flange pressing unit in the plate opening (**b**): 1—die pin, 2—die base, 3—die proper, 4—shaped object (base), 5—holder, 6—holder spring, 7—punch, 8—stop, 9—punch base and spring seat, 10 and 11—fastening elements.

Austenitic steels, e.g., 1.4301, extrusion to other carbon steels, are characterized by a high value of the thermal expansion coefficient and the unit heat capacity (higher by about 30%) [50]. Therefore, the reduction of welding deformations by thermal methods in austenitic steels is ineffective, as it requires a lot of energy (for preliminary heating of the joined elements and heating of the finished product after the welding process-normalization). In the analyzed case, it was found that it is possible to solve the problem by reducing welding deformations by changing the manufacturing process of a sheet element. In accordance with Section 1, an innovative solution has been proposed, i.e., making a hole with a flange by drawing, which will increase the rigidity of the system and introduce a favorable state of compressive stresses, which will reduce the tensile stresses arising in the welding process. For this purpose, the construction of a special tool for extrusion the flange was developed, as shown in Figure 13. The device is equipped with a cone centering the punch in relation to the die before starting the extrusion process.

**Figure 14:**
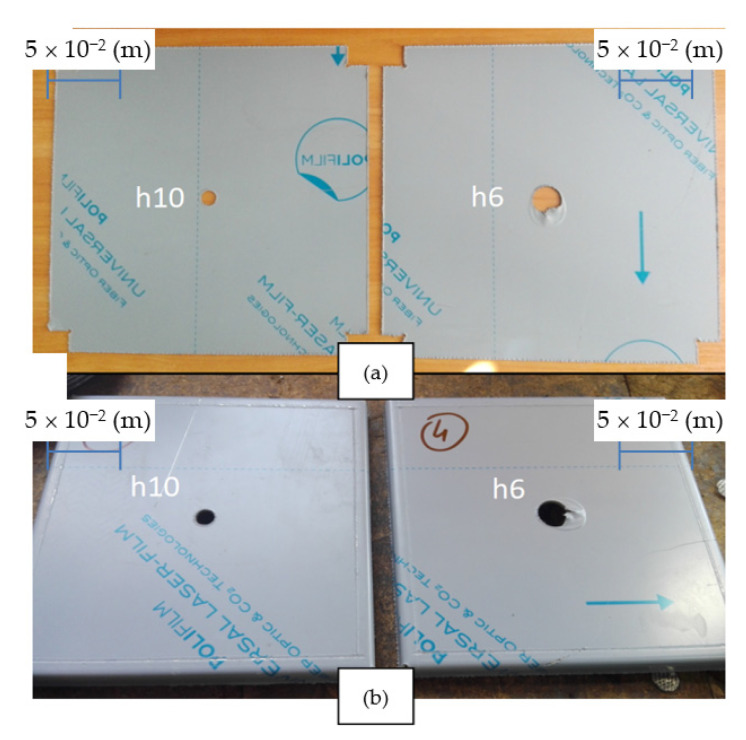
Example research samples (model h6 and model h10) for the flange extrusion process after laser cutting (**a**) and after bending the edges (**b**).

Three test samples were made for each model (h6 and h10 models). Exemplary samples h6 and h10 are shown in Figure 14. Holes with diameters d_0_ = 19.225 × 10^−3^ (m) for the h6 model and d_0_ = 11.225 × 10^−3^ (m) for the h10 model were prepared in the pre-cut laser samples. The edges of the samples were additionally folded.

**Figure 15:**
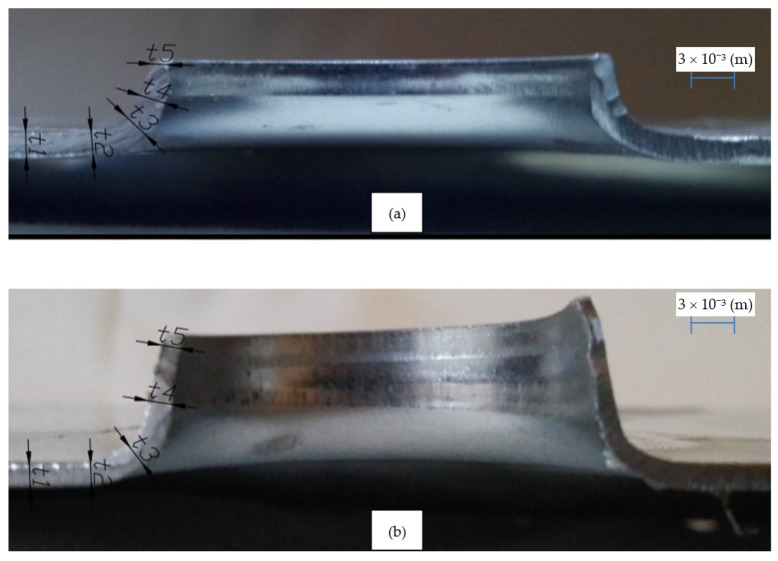
Examples of test samples after the flange extrusion process along with the indicated measurement areas for: h6 model (**a**) and h10 model (**b**).

Before each extrusion process, a lubricant was applied to the contact zones of the punch, die and sample, reducing the frictional forces. Exemplary results of the flange extrusion process are shown in the Figure 15.

**Figure 16:**
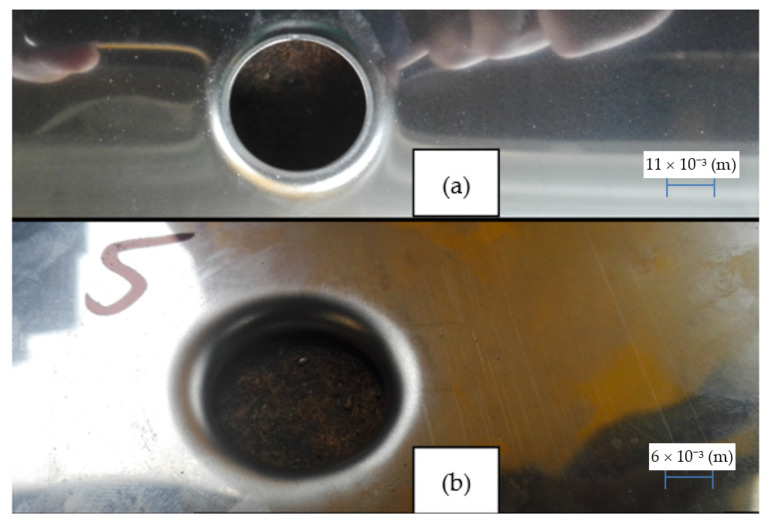
Example research sample after the flange extrusion process for the h6 model: view from above (**a**), internal surface (**b**).

**Figure 17:**
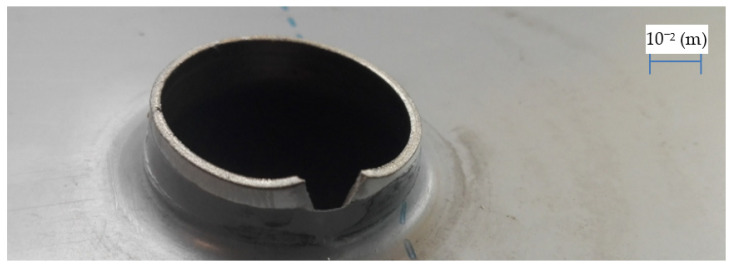
An example of a test sample after the flange extrusion process for the h10 model: side view.

According to FEM calculations, the h6 model proved possible to prepare and a stable high quality of the shaped flange was obtained (Figure 16). On the other hand, the execution of the h10 model as expected turned out to be impossible, as the flange in its upper part was always torn (Figure 17).

After the flange extrusion process for h6 and h10 models, the flange height and wall narrowing were measured. The detailed results are summarized in Table 2, Table 3 and Table 4. The results of the measurements of internal diameters and flange height were made with an accuracy of ±0.02 × 10^−3^ (m). The maximum height for the h10 model flange does not apply to the point where the material structure is broken. 

Table 3 shows the measurement results of the drawpiece wall thickness at characteristic points, as shown in Figure 16. Figure 16 shows the average values of wall thicknesses for both types of drawpieces (h6 and h10 model) and presents the values calculated on the basis of FEM analyzes.

Experimental studies show high compliance with the numerical model of the process in terms of geometric parameters of the obtained flanges on the h6 and h10 models. Only samples made according to the h6 model were used for further experiments. The h10 model has always been damaged and should not be used in further considerations.

## 4. Verification of Numerical Calculations

After the process of drawing a special flange, tests were carried out to weld the socket to the base with a flange for h6 models. The welded joint was made using the TIG method (method 141) (GTAW) in two stages. In the first stage, the elements were joined with a tack weld without wire. Electric current intensity was I_1_ = 90 (A). In the second stage, the actual welding with the wire ϕ 1.2 × 10^−3^ (m) and intensity I_2_ = 40 (A) was performed. The tests were carried out for two different technologies: welding the socket to the base with a hole without a flange (traditional-so far) and welding the socket to the base with a hole with a flange (proposed method). Table 4 shows the detailed welding parameters of the samples.

**Figure 18:**
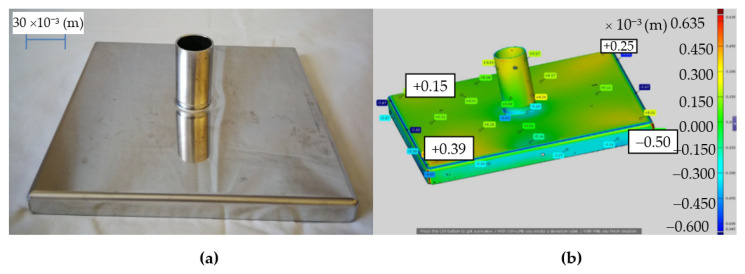
Test sample after TIG welding (method 141) after innovative preparation of a hole with a flange: photo of the real object (**a**), virtual model after 3D scanning with the ATOS III scanner with measurement results (**b**).

The samples after welding were subjected to the measurement of flatness deviations using the ATOS III 3D scanner and the results are summarized in Table 5. Figure 18 shows the experimental sample (model h6) and the virtual model after 3D scanning with the ATOS III scanner for the innovative technology of the method of reducing tank distortions arising during welding the stub-tube made of steel 1.4301 by extrusion the flange, which increases stiffness and introduces circumferential tensile stresses.

The results of calculations and measurements confirm the correctness of the proposed solution to the given problem. The deformation during welding the socket to the tank surface made of 1.4301 steel was significantly reduced. The measurement made with a 3D scanner showed that the maximum flatness deviation (height difference) was Δh ϵ (−0.14; 0.39) × 10^−3^ (m), which is a very small value for this type of construction and technologically acceptable.

## 5. Discussion of Work Results

The presented work pursues two goals: a utilitarian goal and a scientific goal. The utilitarian goal was to develop an inexpensive, fast and effective method of welding stub pipes ϕ 28 × 1.5 × 10^−3^ (m) to the shell of a cylindrical tank with a thickness of t = 1.5 × 10^−3^ (m) without the formation of large shell distortions. The scientific goal was to develop the scientific basis for the reduction of distortions during welding thin-walled parts. The utilitarian goal (special solution) was achieved by implementing the scientific goal. An advanced structural and technological method of reducing distortion in thin-walled welded structures was developed. The essence of the method is to simultaneously increase the stiffness of the system (by introducing design changes) combined with conscious shaping of the stress state. This required the development of a detailed procedure algorithm: finite element analysis of coupled thermal and mechanical effects during welding of thin-walled structures. Universal physical, mathematical, and computer models of mechanical processing combined with heat flow were developed. The developed non-linear equations of motion of objects were solved using FEM. A relationship between effective stress and the flange height determined numerically was developed for sheet EN 1.4301 with a thickness of t = 1.5 × 10^−3^ (m). The presented method is universal and can be used for calculations for the following data: different geometry of the tank (external diameter and wall thickness), different geometry of the socket (diameter and wall thickness), different materials of the tank and the socket, which are characterized by different thermal properties (thermal conductivity, specific heat capacity, density, thermal diffusivity, thermal expansion coefficient, coefficient of convection heat transfer and radiation coefficient) and mechanical (elastic-viscoplastic) during welding (Young’s modulus, hardening modulus, Poisson’s ratio, yield stress), various welding methods (gas, arc), various welding parameters (electrode diameter, welding speed, thermal power, amperage, and voltage at the arc in the case of direct current), different electrode material, cooling conditions, initial and boundary conditions, and preheating. Experimental verification of the solutions obtained on the basis of numerical simulations confirmed their correctness. Some of the results of the experimental research fulfill the utilitarian goal of the work.

## 6. Conclusions

The paper presents typical problems occurring during TIG welding of stubs-pipes for thin-walled large-size tanks made of t = 1.5 × 10^−3^ (m) thick steel 1.4301. The use of traditional technology of welding stubs to the tank shell caused its excessive distortions. The test samples showed a maximum flatness deviation of 11.9 × 10^−3^ (m), which significantly exceeded the permissible standards. A new, innovative method of reducing welding deformations has been developed for the construction of thin-walled large-size tanks with stub pipes by using a new base geometry with an opening with a flange and performing an additional technological operation–flange extrusion. In order to solve this problem, an algorithm of conduct and numerical applications were developed in the Ansys program for analyzes:

- the state of stress and deformations after the welding process for traditional welding,
- the state of stresses and deformations in the process of extrusion an innovative stiffening flange, and
- the state of stresses and deformations after the process of welding the structure with initial stresses caused by shaping the stiffening flange according to the proposed method.

The process of shaping the flange was experimentally tested for two characteristic heights: h = 6 × 10^−3^ (m) and h = 10^−2^ (m). The experimental results confirmed the numerical calculations, which showed that shaping the flange with a height above h > 9 × 10^−3^ (m) may lead to its damage already at the production stage. The results of numerical calculations of the manufacturing process and welding of the stub to the innovative flange were verified experimentally on a designed and built test stand. After the welding process, the deformation of the base was measured with the ATOS III scanner. It was found that the introduction of the proposed design and technological changes favorably reduced the deviations of the base flatness to a maximum value of 0.39 × 10^−3^ (m), which meets the requirements of the standards.

## Figures and Tables

**Table 1:** Physical and mechanical properties of austenitic steel EN 1.4301.

Average coefficient of thermal expansion 293 °K–673 °K (K^−1^)	17.5 × 10^−6^
Thermal conductivity (W/(m·K))	15
Specific heat at 293 °K (J/(kg°K))	500
Electrical specific resistance (Ώ m)	0.73 × 10^−6^
Density (kg/m^3^)	7900
Melting temperature (K)	1673–1723
Yield point Re (Rp_0.2_) min (N/m^2^)	230 × 10^6^
Tensile strength (N/m^2^)	540–750·10^6^
Elongation at break A5 min (%)	45
Hardness HB	215
Modulus of elasticity E (Pa)	193 × 10^6^

**Table 2:** Measurement results of internal diameters and flange height with a measuring accuracy of ± 0.02 × 10^−3^ (m).

Sample Number	d (m)	h_min_ (m)	h_max_ (m)
1—model h10	24.6 × 10^−3^	10.02 × 10^−3^	10.88 × 10^−3^
2—model h10	24.72 × 10^−3^	10.08 × 10^−3^	11.02 × 10^−3^
3—model h10	24.68 × 10^−3^	10.14 × 10^−3^	11.12 × 10^−3^
Average	24.66 × 10^−3^	10.08 × 10^−3^	11.01 × 10^−3^
Standard deviation	0.061 × 10^−3^	0.06 × 10^−3^	0.121 × 10^−3^
4—model h6	24.96 × 10^−3^	6.06 × 10^−3^	6.38 × 10^−3^
5—model h6	24.96 × 10^−3^	6.34 × 10^−3^	6.54 × 10^−3^
6—model h6	24.99 × 10^−3^	6.28 × 10^−3^	6.54 × 10^−3^
Average	24.97 × 10^−3^	6.23 × 10^−3^	6.49 × 10^−3^
Standard deviation	0.014 × 10^−3^	0.12 × 10^−3^	0.075 × 10^−3^

**Table 3:** Measurement results of the wall thickness of the drawpiece with accuracy ±0.02 × 10^−3^ (m) and obtained from the finite element method (FEM) numerical analysis.

Sample Number	t_1_ (m)	t_2_ (m)	t_3_ (m)	t_1_ (m)	t_5_ (m)
1—model h10-experiment	1.52 × 10^−3^	1.44 × 10^−3^	1.22 × 10^−3^	1.10 × 10^−3^	1.06 × 10^−3^
2—model h10-experiment	1.50 × 10^−3^	1.38 × 10^−3^	1.26 × 10^−3^	1.16 × 10^−3^	1.00 × 10^−3^
3—model h10-experiment	1.54 × 10^−3^	1.42 × 10^−3^	1.32 × 10^−3^	1.18 × 10^−3^	1.00 × 10^−3^
Average	1.52 × 10^−3^	1.41 × 10^−3^	1.27 × 10^−3^	1.15 × 10^−3^	1.02 × 10^−3^
Standard deviation	0.02 × 10^−3^	0.03 × 10^−3^	0.05 × 10^−3^	0.04 × 10^−3^	0.03 × 10^−3^
model h10–FEM model	1.50 × 10^−3^	1.44 × 10^−3^	1.42 × 10^−3^	1.21 × 10^−3^	0.99 × 10^−3^
4—model h6-experiment	1.50 × 10^−3^	1.46 × 10^−3^	1.40 × 10^−3^	1.32 × 10^−3^	1.30 × 10^−3^
5—model h6-experiment	1.48 × 10^−3^	1.48 × 10^−3^	1.40 × 10^−3^	1.34 × 10^−3^	1.28 × 10^−3^
6—model h6-experiment	1.50 × 10^−3^	1.46 × 10^−3^	1.40 × 10^−3^	1.36 × 10^−3^	1.30 × 10^−3^
Average	1.49 × 10^−3^	1.47 × 10^−3^	1.40 × 10^−3^	1.34 × 10^−3^	1.29 × 10^−3^
Standard deviation	0.01 × 10^−3^	0.01 × 10^−3^	0	0.02 × 10^−3^	0.01 × 10^−3^
model h6–FEM model	1.50 × 10^−3^	1.49 × 10^−3^	1.45 × 10^−3^	1.32 × 10^−3^	1.29 × 10^−3^

**Table 4:** Welding parameters.

Traditional Technology	New Technology
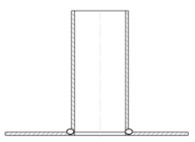	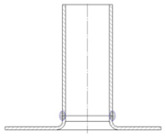
Details of the welding technology	
Material: EN 1.4301	Material: EN 1.4301
Welding method: 141	Welding method: 141
Type of weld: FW-fillet weld	Type of weld: BW-butt weld
Welding position: PH, PB	Welding position: PB
Details:	Details:
Mechanical cleaning-grinding to a metallic sheen	Mechanical cleaning-grinding to a metallic sheen
Tack welds:	Tack welds:
Tack welds-3 every 120°, pipe to the plate connected without a butt joint	Tack welds-3 every 120°, pipe with flange connected without butt joint
Welding parameters:	Welding parameters:
Electric current: I_1_ = 90 (A)	Electric current: I_1_ = 90 (A)
Electric tension: U = 11 (V)	Electric tension: U = 11 (V)
Current type/polarity: DC–	Current type/polarity: DC–
Gas flow: Argon $\dot{V}\;$= 1.2 × 10^−4^ (m^3^·s^−1^)	Gas flow: Argon $\dot{V}$= 1.2 × 10^−4^ (m^3^·s^−1^)
Without binder	Without binder
Proper welding:	Proper welding:
Electric current: I_1_ = 40 (A)	Electric current: I_1_ = 40 (A)
Electric tension: U = 13 (V)	Electric tension: U = 13 (V)
Current type/polarity: DC–	Current type/polarity: DC–
Gas flow: Argon $\dot{V\;}$= 1.2 × 10^−4^ (m^3^·s^−1^)	Gas flow: Argon $\dot{V}\;$= 1.2 × 10^−4^ (m^3^·s^−1^)
Binder: wire WMoSi diameter ϕ =1.2 × 10^−3^ (m)	Binder: wire WMoSi diameter ϕ =1.2 × 10^−3^ (m)

**Table 5:** Summary of the measurement results of the flatness deviation values of two scanned elements.

Description of the Technology	Sample Flatness Deviation [m]					
	1		2		3	
	min	max	min	max	min	max
Welding directly into the hole	−0.71 × 10^−3^	+11.09 × 10^−3^	−0.63 × 10^−3^	+9.19 × 10^−3^	−0.55 × 10^−3^	+10.56 × 10^−3^
Welding to an innovative flange	−0.14 × 10^−3^	+0.39 × 10^−3^	−0.12 × 10^−3^	+0.13 × 10^−3^	−0.04 × 10^−3^	+0.29 × 10^−3^

## Data Availability

No new data were created or analyzed in this study. Data sharing is not applicable to this article.
